# Accurate Alternative Measurements for Female Lifetime Reproductive Success in *Drosophila melanogaster*

**DOI:** 10.1371/journal.pone.0116679

**Published:** 2015-06-30

**Authors:** Trinh T. X. Nguyen, Amanda J. Moehring

**Affiliations:** Department of Biology, Western University, London, ON, Canada; CNRS, FRANCE

## Abstract

Fitness is an individual’s ability to survive and reproduce, and is an important concept in evolutionary biology. However, accurately measuring fitness is often difficult, and appropriate fitness surrogates need to be identified. Lifetime reproductive success, the total progeny an organism can produce in their lifetime, is thought to be a suitable proxy for fitness, but the measure of an organism’s reproductive output across a lifetime can be difficult or impossible to obtain. Here we demonstrate that the short-term measure of reproductive success across five days provides a reasonable prediction of an individual's total lifetime reproductive success in *Drosophila melanogaster*. However, the lifetime reproductive success of a female that has only mated once is not correlated to the lifetime reproductive success of a female that is allowed to mate multiple times, demonstrating that these measures should not serve as surrogates nor be used to make inferences about one another.

## Introduction

An organism’s success in the presence of selection is defined by their fitness [1–4]. While the idea of fitness as the production of offspring, who are in turn successful in producing offspring, is conceptually easy to understand, there has been debate as to the appropriate way to measure fitness within a laboratory setting [5–7]. These measurements must be a phenotype that is able to be scored in a reasonable manner, yet accurately capture the essence of an organism’s fitness. In an attempt to measure fitness, studies often measure more tractable surrogates of fitness such as body size, survivability, viability, growth rate, mating success, longevity, fecundity, and fertility (e.g, [8–10]). Of these alternative measurements, the number of offspring an individual produces over its lifetime (lifetime reproductive success) is generally considered an acceptable estimate of fitness [2,6,11]. However, for species with multiple reproductive cycles, long generation times, or large numbers of offspring, lifetime reproductive success is often difficult and time-consuming to measure. Studies therefore often measure reproductive success over only a subset of an organism’s lifespan as an approximation of lifetime reproductive success [12–18]. However, using short term reproductive success as a measure of fitness can potentially be inaccurate if organisms vary in their rates of offspring production, such as through a trade-off in quantity of early *vs*. late lifetime reproductive output.

*Drosophila melanogaster* is a model organism that is often used in studies with a fitness component (e.g., [19–22]). Under unlimited conditions of food and access to mates, a female will produce an average total of 615 offspring throughout her lifetime [23], which is approximately 90 days at 21 degrees Celsius for wild-type *D*. *melanogaster* [24]. The long life expectancy and high productivity of *D*. *melanogaster* make it time-consuming to measure the total lifetime reproductive success, particularly when sample sizes are large, and thus surrogate measures of fitness are usually used in this species. Measuring reproductive output over a much shorter time span or after only a single mating could potentially serve as accurate proxies for lifetime reproductive success, but a direct test of the relationship between these alternative measures and lifetime reproductive success has not been conducted for this widely-used model species. Here, we use multiply-mated females from ten isofemale lines of *D*. *melanogaster* to determine if a female’s short-term reproductive output (after one day and/or seven days) can accurately predict lifetime offspring production. We also determined the optimal number of days to measure reproductive output in order to achieve the strongest correlation with lifetime reproductive success using the fewest number of measurements. We then compared lifetime reproductive success of multiply-mated females to that of singly-mated females to assess whether a female's reproductive output from a single mating, which is less cumbersome to measure, is indicative of her output after multiple matings, which is more representative of a female's mating status in the wild.

## Materials and Methods

Ten isofemale lines of *D*. *melanogaster*, collected from the wild in Sudbury, Ontario Canada, in 2011, were generously provided by T. Merritt. Flies were maintained in the laboratory on standard cornmeal agar media (Bloomington *Drosophila* Stock Center, Indiana) in 8-dram vials on a 14:10 light-dark cycle, at 24°C and approximately 75% relative humidity. Males and females were separated upon eclosion (to ensure virginity), aged four to six days, and then placed in single mating pairs within a vial. Additional males were collected at the same time but left unmated; these aged males were used as replacements for similarly-aged males who died.

For multiply-mated females, pairs were kept together throughout the female’s lifetime, allowing for remating. The ten isofemale lines were mated in a full-factorial diallel cross, resulting in 100 mating pairs, each with four replicates. Mated pairs were checked daily and dead males were replaced with a male of similar age. Mating pairs were transferred into a new vial after one day, transferred again after an additional six days (seven days after initial mating), and then every seven days thereafter. The measure of offspring from the initial vial is the reproductive output from one day (the number of offspring that eclose from the total eggs laid in one day), the measure of offspring from the first vial plus the second vial is the reproductive output after seven days (the number of offspring that eclose from the total eggs laid in 7 days), and the measure of the offspring produced from all of the vials in a female’s lifetime is the lifetime reproductive success. The number of offspring eclosing from each vial was scored daily, up until 16–17 days after the last egg was laid or the female died, ensuring enough time for all larvae to emerge and that all offspring that were produced were scored. Since offspring eclosion was recorded daily, the total daily eclosion and the total daily cumulative eclosion measures were analyzed. The total daily eclosion measures consist of the total number of eclosions that occurred each day after the first eclosion, regardless of when the eggs were laid. The total daily eclosion measures may differ from the eclosion measures from the one day and 7 day block (previously stated) since these were scored based on the day the eggs were laid rather than the day of eclosion, and variation in larval developmental times could cause these values to differ. Any female that did not produce any larvae, indicating that mating did not occur or that individuals were sterile, was removed from the data set. We note that the lifetime reproductive success of females measured here may not be representative of the values that may occur in nature, as these laboratory females are supplied with unlimited food and mating opportunities, and are not subjected to predation or competition.

**Fig 1:**
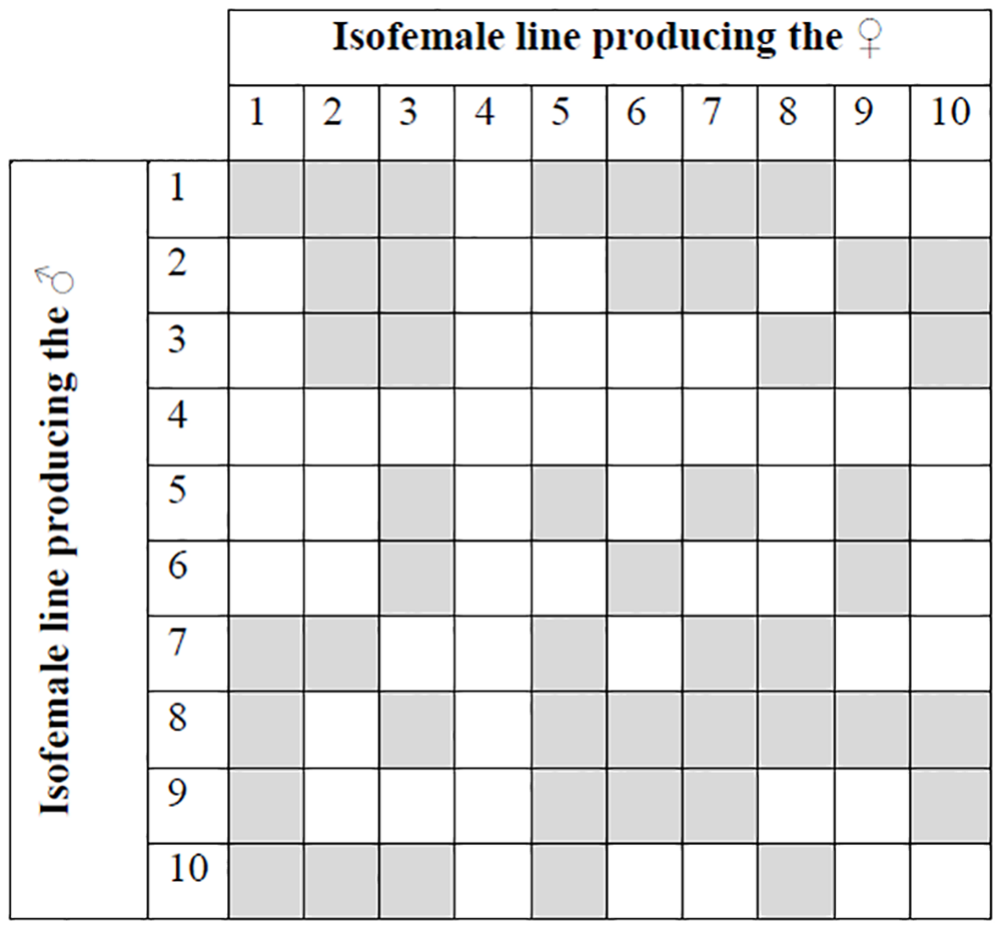
Isofemale line combinations that were assayed. Combinations that were mated in singly-mated crosses are shaded (see Methods). All combinations (shaded and unshaded) were used in the multiply-mated crosses.

For singly mated females, mating assays were performed with a single male and female in each vial and males were removed after mating; unmated flies were discarded. Isofemale line combinations that were mated are shown in Fig 1 for a total of 47 mating pairs, each with 20 replicates. Females were transferred into a new vial every seven days and the number of offspring eclosing from each vial was scored in a similar manner as above.

To determine whether early short term reproductive success (one day and seven days) could be used to predict lifetime reproductive success, a linear model (LM) was performed using lifetime reproductive success as the response variable and short term reproductive success (one day or seven days) as the predictors. A similar LM was used to determine whether early reproductive success could be used to predict late reproductive success. Late reproductive success was calculated by excluding early reproductive success measures from lifetime reproductive success. For comparison to a previous study [25], a LM with quasipoisson distribution was performed using a short term reproductive success window of 7 days after approximately 30 days of offspring emergence. The between and within variation in isofemale lines for lifetime reproductive success of singly mated females was analyzed in a two-way ANOVA with a Tukey's post hoc using female line and male line as factors. To compare singly and multiply mated isofemale line crosses, a linear mixed model (LMM) was performed using the average multiply mated lifetime reproductive success for each isofemale line combination as the response variable and the corresponding isofemale line combination average of singly mated lifetime reproductive success as the predictor variable, along with female line and male line as random factors. All analysis were performed in R 3.0.3 [26]. All raw data is available in S1–S4 Tables.

## Results

**Fig 2:**
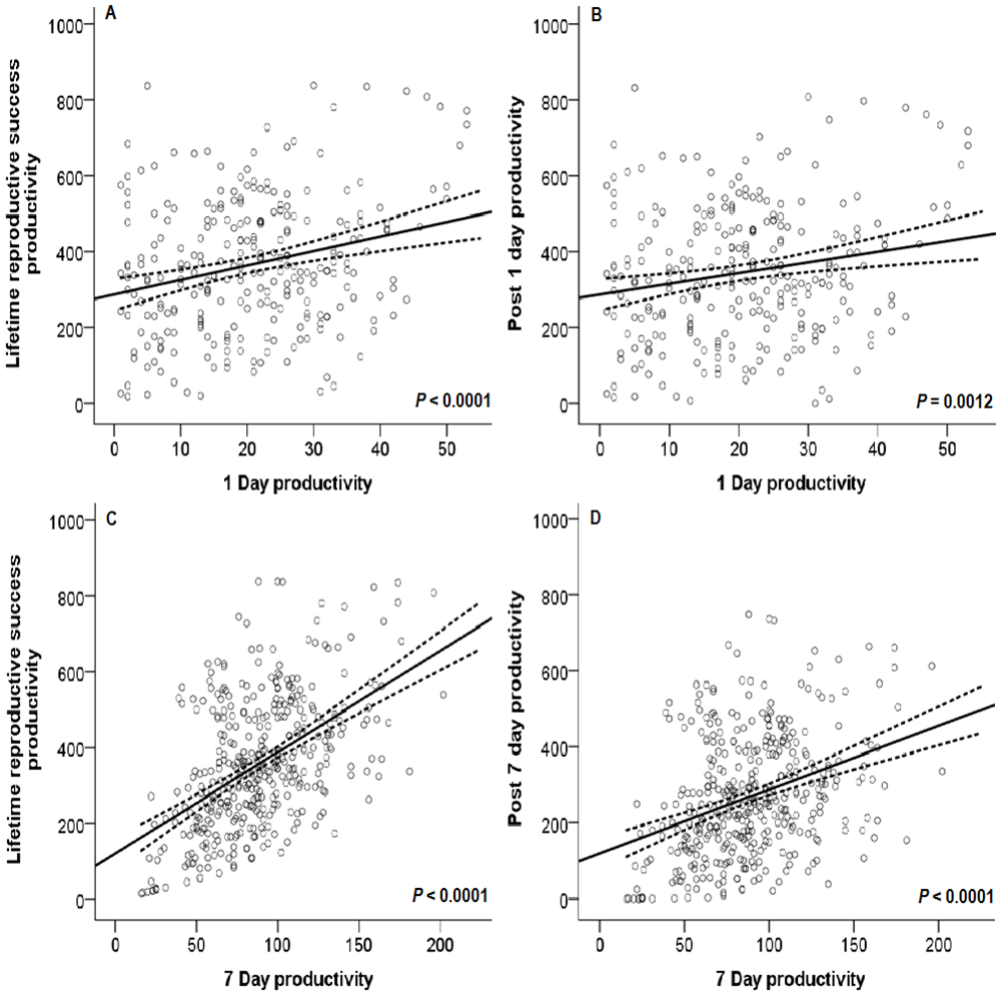
Regression of early short-term reproductive outputs on lifetime reproductive success. Early reproductive success is defined by the number of offspring that eclosed from eggs laid in the first day (A, B) or the first seven days (C, D). These values were compared to a total lifetime reproductive success response variable that either included values of short-term reproductive success (A, C) or that excluded the short-term reproductive success values of one day (B) or seven days (D). Dashed lines represent the 95% CI.

Early, one-day reproductive success can predict lifetime reproductive success (Fig 2A; Estimate = 3.8386 ± 0.8717 S.E., F _(1, 267)_ = 19.39, *P* < 0.0001, R^2^ = 0.0642). Similarly, one-day reproductive success can predict late (older than 1 day) reproductive success (Fig 2B; Estimate = 2.8386 ± 0.8717 S.E., F _(1, 267)_ = 10.60, *P* = 0.0012. R^2^ = 0.0346). While these measures are predictive, they only explain 6.4% of the variation in lifetime measurement. This is likely because pairs of flies were not scored for the timing of mating, and were simply removed 24 hours after being paired. Fly pairs therefore could have mated at any time within the 24 hours, and females who mated at the end of this time period would have laid very few fertilized eggs.

Similarly, early seven-day reproductive success is a strong predictor for lifetime reproductive success (Fig 2C; Estimate = 2.6790 ± 0.2250 S.E., F _(1, 398)_ = 141.8, *P* <0.0001, R^2^ = 0.2608) and can predict late reproductive success (older than 7 days) (Fig 2D; Estimate = 1.6790 ± 0.2250 S.E, F _(1, 398)_ = 55.68, *P* < 0.0001, R^2^ = 0.1205). The mean one-day reproductive output is 20.72 (19.28–22.17 95% CI, values ranging from 1–53), mean seven-day reproductive output is 84.38 (80.68–88.07 95% CI, values ranging from 16–165), and mean lifetime reproductive output is 345.63 (325.72–365.54 95% CI, values ranging from 16–838).

**Fig 3:**
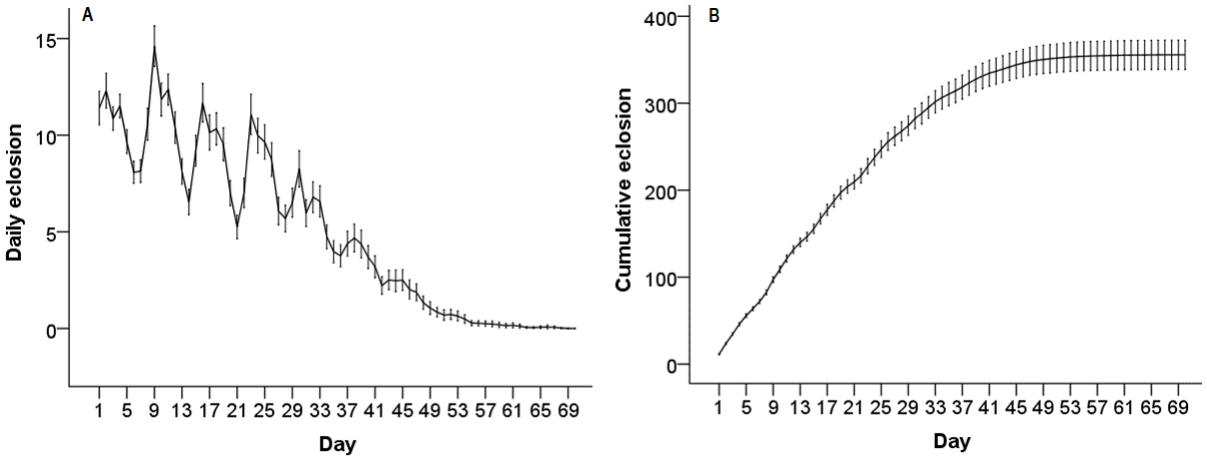
Daily eclosion rates. **(A)** Mean daily eclosion, measured as the total number of offspring eclosing on each day. **(B)** Mean cumulative eclosion per day. ‘Day 1’ is the first day that offspring eclosed. Error bars represent the 95% CI.

There is a consistently high rate of offspring eclosion up until approximately day 25 after the first offspring ecloses, with peak eclosion at approximately day 10 (Fig 3). Interestingly, there are fluctuations in eclosion rates on an approximately 7 day cycle (Fig 3A). This may correspond with the timing of tipping the females to new vials, but since the correspondence of fly tipping with eclosion was not scored we are unable to assess this directly. However, this is unlikely to be due to food limitation since we see the cycle even when the peak number of offspring eclosing is relatively low (e.g. days 29–36 and 37–43, Fig 3A), suggesting that the cycle may be due to inducing increased egg laying upon transfer to a new food source. When evaluating the minimum window of early reproduction that could be measured as a proxy for lifetime reproductive success (LRS), even the first day of eclosion has a significant correlation with LRS (Table 1). However, as expected, correlation values increase as more days are scored, with the greatest gains in R^2^ occurring up to day 5 (Table 1).

**Table 1:** Predicting total lifetime reproductive success from daily cumulative eclosion.

Parameter^1^	Estimate (SE)^2^	F_(1, 398)_	*P*—value	R^2^
1 Day	3.8686 (0.9579)	16.31	6.45e-05	0.0369
2 Day	3.5868 (0.6783)	27.96	2.045e-07	0.0633
3 Day	3.9042 (0.5549)	49.50	8.704e-12	0.1084
4 Day	3.7284 (0.4545)	67.30	3.259e-15	0.1425
5 Day	3.7665 (0.3953)	90.80	<2.2e-16	0.1837
6 Day	3.3235 (0.3534)	88.46	<2.2e-16	0.1798
7 Day	3.3636 (0.3129)	115.50	<2.2e-16	0.2230
8 Day	3.2656 (0.2654)	151.40	<2.2e-16	0.2737
9 Day	2.8440 (0.2106)	182.30	<2.2e-16	0.3124
10 Day	2.6479 (0.1869)	200.70	<2.2e-16	0.3335

^1^ The number of cumulative days after the day of first eclosion

^2^ Estimated via a linear model.

**Fig 4:**
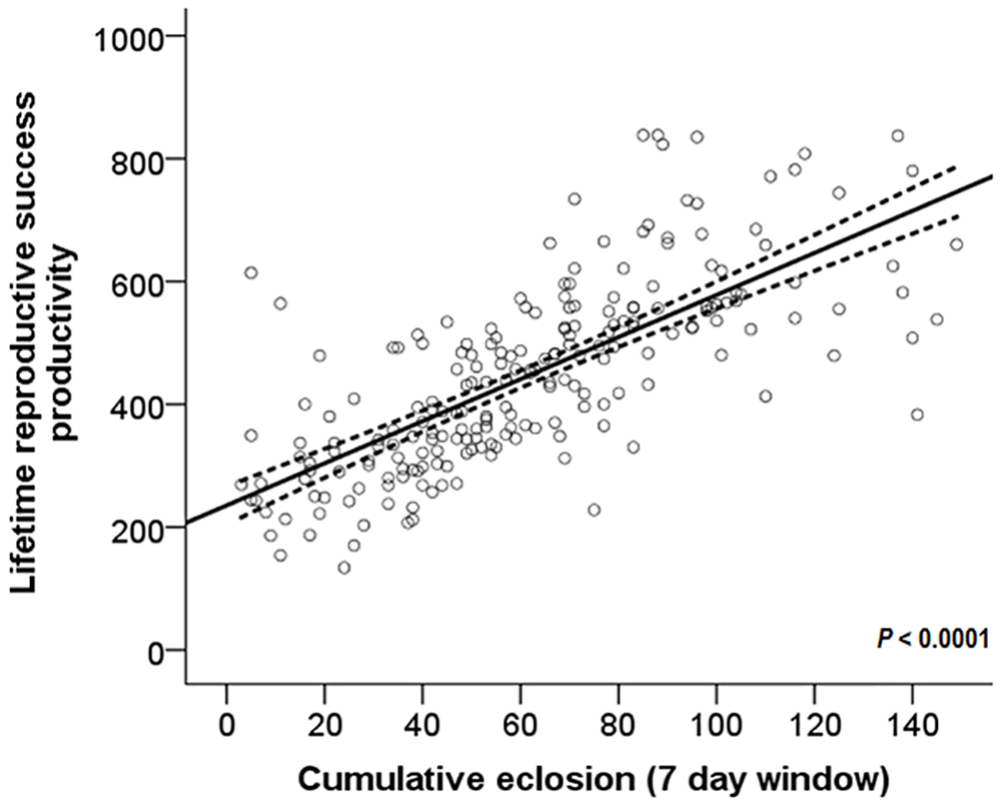
Regression of late short term reproductive output on lifetime reproductive success. Late short-term reproductive success was measured as the total number of offspring eclosing during a seven day window after females were approximately 30 days old. Dashed lines represent the 95% CI.

**Fig 5:**
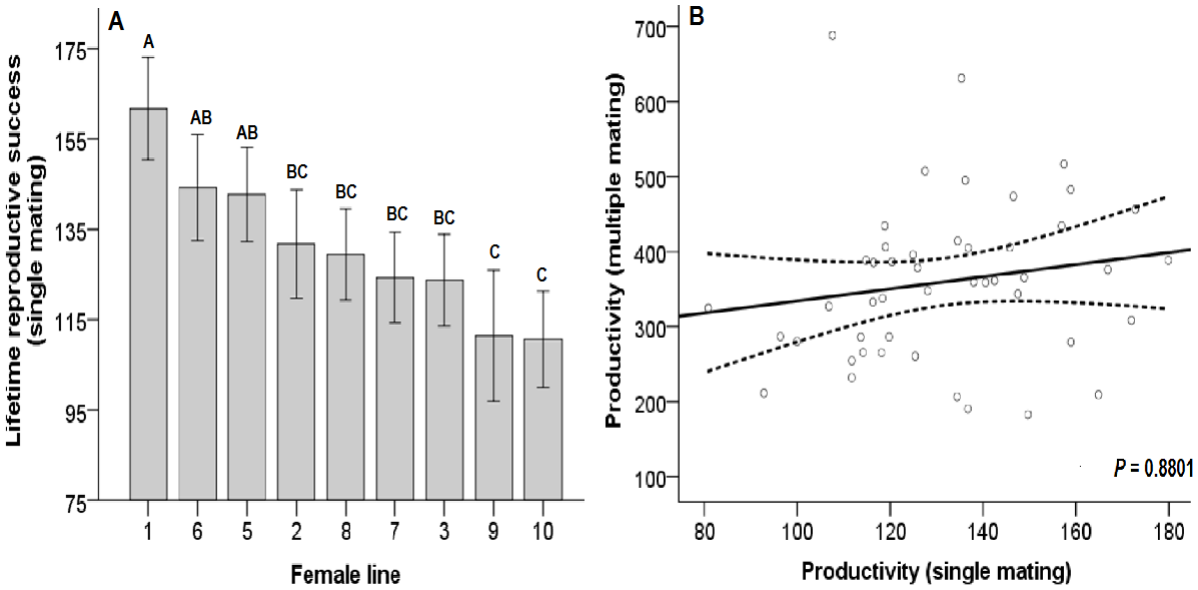
Reproductive success by line and by mating level. **(A)** Variation of lifetime reproductive success of singly mated females separated by female line. Columns with the same letters are not significantly different. Error bars represent the 95% CI. **(B)** Regression of mean productivity of females with multiple matings on productivity of singly mated females. Dashed lines represent the 95% CI.

A seven-day reproductive success window for older females (after approximately 30 days of offspring emergence) is a strong predictor for total lifetime reproductive success (Fig 4; Estimate = 0.0072 ± 0.0004 S.E., t _(211)_ = 14.88, *P* <0.0001, pseudo R^2^ = 0.5083). The two-way ANOVA revealed a significant female line effect (Fig 5A; F _(8, 866)_ = 8.2960, *P* < 0.0001) and significant male line effect (F _(8, 866)_ = 7.7590, *P* < 0.0001) for the lifetime reproductive success of singly-mated females. No significant interaction was detected (F _(30, 836)_ = 0.7170, *P* = 0.8680). Of note, the productivity from singly-mated flies was not a significant variable in determining productivity from multiply-mated flies (Fig 5B; χ^2^_(1)_ = 0.0228, *P* = 0.8801).

## Discussion

Early, short-term reproductive success measures of one or seven days can accurately predict both lifetime reproductive success and late reproductive success in *D*. *melanogaster* (Fig 2). However, seven days of reproductive success is more accurate as an indicator and can explain more of the variation in lifetime reproductive success than the very short-term measure of one day. Similarly, a short term reproductive success measurement of a seven day window in older females is highly significant (*P*<0.0001) in predicting their lifetime reproductive success (Fig 4). Our results concur with those of Pekkala *et al*. (2011) who showed low but significant correlations of short-term measures (2 day, 4 day, and 10 day windows) of offspring production and lifetime reproductive success for young females in *Drosophila littoralis* [25]. Our results also concur with their findings in older females, where there is a high correlation between offspring production measured during a brief window later in life and lifetime reproductive success (correlation up to 0.83). This comparison of similar studies in different species demonstrates that some aspects of reproductive success may show a consistent trend across *Drosophila*; however caution should still be used in applying these results to other species of *Drosophila*.

Although it is evident the longer the initial measures of reproductive success, the more accurately it can predict lifetime reproductive success, the question remains is how many days in early life is optimum to predict lifetime reproductive success. Although a measure of one day of eclosion is statistically significant, it only explains 6.4% of the variation in lifetime reproductive success. According to our results, it appears the cumulative eclosion measure of the initial 5 days in early life is optimal, explaining 18.37% of the variation in lifetime reproductive success, with minimal increase in predictive power at day 6 (Table 1). Therefore, studies involving lifetime reproductive success measures may obtain an optimal balance of accuracy *vs*. labor by measuring the initial reproductive success of the first 5 days of offspring eclosion.

The regression of early short term reproductive success (1 day or 7 days) on later reproductive success (>1 day or >7 days) shows a positive correlation (Fig 2B and 2D). Therefore, having an initially high reproductive output does not come with a reproductive trade-off cost later in life, counter to what would be expected if antagonistic pleiotropy was occurring [27,28]. Similar positive pleiotropic effects are seen in the bedbug, *Cimex lectularius*, where higher ejaculate doses both increase reproductive rates and delays female reproductive senescence [29]. Interestingly, the peak daily eclosion does not occur from eggs laid in very early life, counter to expected. Instead, peak eclosion numbers occur from eggs laid later in life, approximately on day 10 of eclosion (eggs laid when females are approximately 14–16 days old), which is shortly after females would be expected to regain receptivity towards a courting male and accept a second mating (at ~8–9 days old; [30]). This suggests that peak female fecundity may not occur until females have mated a second time.

Although very short term reproductive success values from one day are not strongly predictive of lifetime reproductive success in the laboratory, they may be an accurate fitness measure in natural environments, although this likely depends on the species being examined. The average life expectancy in the wild is approximately three days for domesticate species of *Drosophila* (e.g. *D*. *melanogaster*, *D*. *simulans*, *D*. *immigrans*, etc; [31]), approximately 6 days for *D*. *serrata* [32], and approximately seven days for *D*. *mercatorum* [33]. Hence, the reproductive output from a shorter time span may more accurately reflect the biological fitness of an organism, even if it does not reflect the total reproductive output possible in the laboratory, if that longer lifespan is not realized in the wild.

Significant female line effects for the lifetime reproductive success of singly mated females indicate that the fecundity of a singly mated female can predict the fecundity of another singly mated female from the same isofemale line, regardless of who the female mates with. Therefore, a similar relationship could be expected with singly and multiply mated females. However, contrary to this, the productivity of from a single mating does not predict lifetime productivity when allowing for remating in *D*. *melanogaster*. The relationship (or lack thereof) between the reproductive output of single and multiple matings is not universal across species: for example, in the Bruchid beetle, *Callosobruchus maculatus* (Coleoptera: Bruchidae), there was no difference in fecundity between singly mated females and females who were confined to a single male during her lifetime, which allowed for remating [34]. In *D*. *melanogaster*, the lack of a relationship between single and multiply-mated females is likely due to sperm limitation (the male’s contribution) in the former case and egg production limitation (the female’s contribution) in the latter case. Similar to our results, multiply-mated *D*. *pseudoobscura* females had a higher productivity than singly-mated females, suggesting that singly-mated females are sperm limited [12]. However, this sperm limitation has only a moderate effect on productivity in our study since singly mated females had 82% of the productivity of multiply mated females [33].

Our results, together with Pekkala *et al*. (2011) suggest that one or two day reproductive measurements are appropriate indicators of an individual's total lifetime reproductive success in *Drosophila*. Short-term measurements of the initial seven days of offspring production in young females can, however, explain more variation (26%) in total lifetime reproductive success in *D*. *melanogaster*. It is important to note that this significant short term measure of reproductive success applies to multiply-mated females. There was no correlation between singly and multiply mated females, and thus these measures should not be used to make inferences about each other. However, within both *D*. *melanogaster* (presented here) and *D*. *littoralis* [25], it appears that a well-timed window measurement of seven days in older females is significantly correlated to lifetime reproductive success, and thus this measure may also potentially serve as an accurate proxy across the *Drosophila* genus [25].

## Supporting Information

S1 Table Offspring produced from one day of egg production by multiply-mated females. (XLSX)

S2 Table Offspring produced from 7 days of egg production by multiply-mated females. (XLSX)

S3 Table Lifetime reproductive success of multiply-mated females. (XLSX)

S4 Table Lifetime reproductive success of singly-mated females. (XLSX)
